# Pain management after hallux valgus repair surgery: an updated systematic review and procedure-specific postoperative pain management (PROSPECT) recommendations

**DOI:** 10.1097/EJA.0000000000002302

**Published:** 2025-10-21

**Authors:** Melissa Wust, Neel Desai, Girish P. Joshi, Narinder Rawal, Marc Van de Velde, Eleni Moka, Jolanda Elmers, Eric Albrecht

**Affiliations:** From the Department of Anaesthesiology, University Hospital of Lausanne and University of Lausanne, Lausanne, Switzerland (MW), Guy's and St Thomas’ NHS Foundation Trust, London, UK (ND), the Department of Anesthesiology and Pain Management, University of Texas Southwestern Medical Center, Dallas, Texas, USA (GPJ), Örebro University, Örebro, Sweden (NR), Department of Cardiovascular Sciences, KU Leuven and Department of Anaesthesiology, UZ Leuven, Leuven, Belgium (MvDeV), the Department of Anaesthesia, Creta Interclinic Hospital, Hellenic Healthcare Group (HHG), Heraklion-Crete, Greece (EM), The Medical librarian, Medical Library, University Hospital of Lausanne and University of Lausanne, Lausanne, Switzerland (JE), Program Director of Regional Anaesthesia and Clinical Research, Department of Anesthesiology, University Hospital of Lausanne and University of Lausanne, Lausanne, Switzerland (EA)

## Abstract

**BACKGROUND:**

Hallux valgus repair surgery is associated with moderate-to-severe postoperative pain. The aim of this systematic review was to assess the available literature and update previous PROSPECT (PROcedure SPECific Postoperative Pain ManagemenT) recommendations for optimal pain management after hallux valgus repair surgery.

**METHODS:**

A systematic review utilising PROSPECT methodology was performed. Randomised controlled trials and systematic reviews published in the English language from January 1, 2019 to November 19, 2024 that assessed postoperative pain using analgesic, anaesthetic and surgical interventions were identified from CENTRAL, CINAHL, EMBASE, MEDLINE and Web of Science.

**RESULTS:**

Of the 375 articles identified, 17 RCTs and 7 systematic reviews/meta-analyses met our inclusion criteria (total: 24 publications). Interventions that improved postoperative pain relief included: paracetamol and nonsteroidal anti-inflammatory drugs or cyclooxygenase-2 selective inhibitors; dexamethasone; ankle block and, as an alternative, local anaesthetic wound infiltration; and minimally invasive surgery or percutaneous osteotomy. Insufficient evidence was found for the use of perineural magnesium or liposomal bupivacaine. No evidence was found for continuous popliteal sciatic nerve block or for the use of the plantar compartment nerve block.

**DISCUSSION:**

This review provides an update to the previous guidelines written by the PROSPECT group: there is one important change, minimally invasive surgery or percutaneous osteotomy is recommended over open osteotomy. Contemporary publications confirm the analgesic effects of ankle block as a first-choice modality with wound infiltration as an alternative. In addition, the analgesic regimen for hallux valgus repair should include, in the absence of contraindication, paracetamol and a nonsteroidal anti-inflammatory drug or cyclooxygenase-2 selective inhibitor administered preoperatively or intra-operatively and continued postoperatively, along with systemic dexamethasone, and postoperative opioids for rescue analgesia.

**KEY POINTS**

- This systematic review provides PROSPECT updated recommendations for pain management after hallux valgus repair surgery.
- The analgesic regimen should include, in the absence of contraindication, paracetamol and a nonsteroidal anti-inflammatory drug or cyclo-oxygenase-2 selective inhibitor administered peri-operatively and continued postoperatively, along with systemic dexamethasone, and postoperative opioids for rescue analgesia.
- Interventions that improved postoperative pain relief include ankle block and, as an alternative, local anaesthetic wound infiltration; and minimally invasive surgery or percutaneous osteotomy.

## Introduction

Pain after hallux valgus repair surgery is moderate to severe.^1^ Many pharmacological treatments, anaesthetic strategies and surgical techniques have been investigated to provide the best possible postoperative analgesia. Previous PROSPECT (PROcedure-SPEcific Pain ManagemenT) guidelines for pain management were published in 2020.^2^ The PROSPECT Working Group is a collaboration of anaesthesiologists and surgeons working to formulate procedure-specific recommendations for pain management after common surgical procedures. The recommendations are based on the procedure-specific literature review of randomised controlled trials (RCTs) and systematic reviews. A special feature of PROSPECT recommendations is that the methodology considers clinical practice, efficacy and adverse effects of analgesic techniques and is hence holistic in nature.^3^

Given that multiple trials have been published since the last PROSPECT recommendations on hallux valgus repair surgery, we decided to perform a review of the contemporary literature to ensure that the evidence base for these international recommendations remained up to date. The objective of this review was to systematically assess the available literature on pain management following hallux valgus repair surgery. Postoperative pain outcomes, that is pain scores and analgesic requirements, were the primary outcomes. Other outcomes, including the incidence of adverse effects, were also evaluated, and the limitations of the data were also reviewed. The aim was to update the recommendations for pain management after hallux valgus repair.

## Methodology

In the conduct of the review, we adhered to the previously described PROSPECT methodology.^3^ The systematic review was registered on PROSPERO (CRD420251013056, registered on March 27, 2025).

The following databases were specifically searched from January 1, 2019 to November 19, 2024 for any RCTs that investigated any intervention for hallux valgus repair surgery and reported pain scores and which were published in the English language: the Cumulative Index to Nursing and Allied Health Literature (CINAHL); Cochrane Central Register of Controlled Clinical Trials (CENTRAL); Excerpta Medica database (EMBASE); the U.S. National Library of Medicine Database (MEDLINE); and the Web of Science. The applied intervention search terms and keywords included: hallux valgus; bunionectomy; metatarsal osteotomy; pain; and analgesia. Deduplication of the retrieved records was done manually. Population limits were then placed including Clinical trials OR Random allocation OR Therapeutic use. Details of the search strategy are provided in appendix 1 (supplementary data). In addition to RCTs, review articles (e.g. systematic reviews, meta-analyses and umbrella reviews) assessing analgesic interventions specific to pain management for the selected procedure will also be identified. The RCTs within these publications will be scrutinised, and those identified for inclusion will be analysed critically according to PROSPECT methodology. We excluded any study that described a phase II investigation of a drug that was unlicenced at the time of this review, and trials which compared different local anaesthetics; or doses, concentrations and volumes of the same analgesic with no control group.

Data extraction and data analysis as well as quality assessment followed the PROSPECT methodology.^3^ Pain intensity scores were used as the primary outcome measure. A change of more than 1 out of 10 on the numerical rating score (NRS) or 10 mm on a 100 mm visual analogue scale (VAS) was defined as clinically relevant.^4^ The effectiveness of each intervention for every outcome was evaluated by assessing the differences reported between treatment arms in each trial. Meta-analysis was not performed due to heterogeneity in trial design and result reporting, restricting the possibility of pooled analysis. We made recommendations according to PROSPECT methodology, based mainly on critical evaluation of included RCTs and, if deemed appropriate, on conclusions of review articles.^3^ The proposed recommendations were sent to the PROSPECT Working Group for review and comments, and a modified Delphi approach was then used as previously described until consensus was achieved. The lead authors then drafted the final document which was ultimately approved by the PROSPECT Working Group.

## Results

**Fig. 1:**
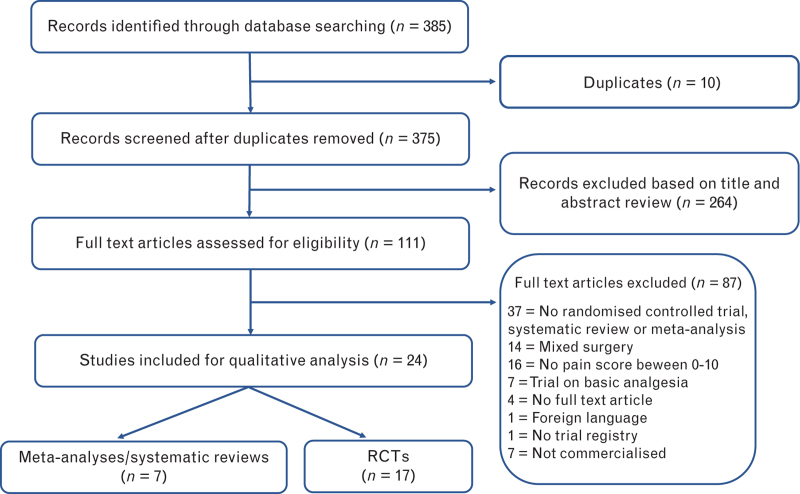
PRISMA flow diagram summarising the retrieved, included and excluded randomised controlled trials and systematic reviews.

**Fig. 2:**
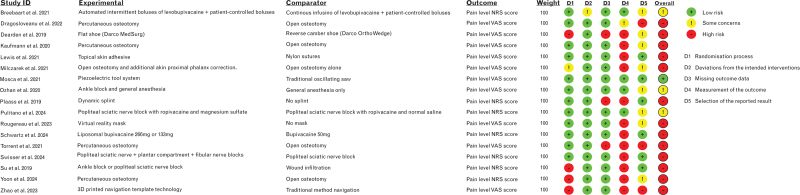
Risk of bias 2 evaluation of included trials using the Cochrane Collaboration's tool.

From the 375 records retrieved from the literature search, 17 RCTs^5–21^ and seven systematic reviews/meta-analysis^22–28^ were finally included (Fig. 1). Noteworthily, no new trial was identified from the meta-analyses. Supplementary table S1 presents a summary of the key findings of trials assessing interventions that were recommended, while supplementary table S2 provides a summary of the key results of trials evaluating interventions which were not recommended. Figure 2 presents the quality assessment of included trials using the Cochrane Risk of Bias Tool 2. Supplementary table S3 lists the excluded trials and the reasons for exclusion.

No new trials were identified from the systemic reviews/meta-analyses. PRISMA, Preferred Reporting Items for Systematic Reviews and Meta-Analyses.

Green, low risk; yellow, some concerns; red, high risk. D1, randomisation process; D2, deviations from the intended interventions; D3, missing outcome data; D4, measurement of the outcome; D5, selection of the reported result.

### Regional anaesthesia

Three publications examined the analgesic efficacy of an ankle block.^12,18,27^ One meta-analysis of 5 articles (459 patients; 2 articles included for the current recommendations, 3 excluded as they were published before 2019), concluded that the postoperative analgesic efficacy of the ankle and popliteal sciatic nerve blocks are similar.^27^ Further, it was found that the ankle block decreased the pain score at 6 h compared with peri-incisional infiltration.^27^ The superiority of the ankle block over control or peri-incisional infiltration was recently confirmed by two prospective trials on 110 patients^12^ and 75 patients.^18^ In these two trials, basic analgesics (paracetamol and a nonsteroidal anti-inflammatory drug or cyclooxygenase-2 selective inhibitor) were routinely prescribed. Indeed, compared with no ankle block, Ozhan *et al.* found a reduction in the pain score of 2.9 at 12 h with ankle block,^12^ and, relative to peri-incisional infiltration or no block, Su *et al.* showed a reduction in the pain score of >1.5 in the recovery room and at 6 h.^18^ Both trials also uncovered the potential of ankle block to reduce postoperative opioid consumption.^12,18^

Four other trials investigated the addition of different interventions along with the popliteal sciatic nerve block.^5,14,16,17^ In one trial, that included 59 patients, the addition of a plantar compartment nerve block and fibular nerve block to a popliteal sciatic nerve block did not provide any analgesic benefit.^17^ Likewise, there was no difference in the mode of local anaesthetic administration through the perineural catheter in continuous popliteal sciatic nerve block. The pain score and opioid consumption were similar regardless of whether patients had, on top of self-administered boluses, a continuous infusion of 5 ml h^−1^ or automated intermittent boluses of 9.8 ml every 2 h.^5^ On the other hand, in another trial that included 49 patients, the addition of perineural magnesium 200 mg to the local anaesthetic in popliteal sciatic nerve block decreased the pain score by 4 between 12 h and 24 h. Moreover, opioid consumption was reported to be reduced, although details were not provided.^14^ Finally, in one trial, that included 273 patients, no difference was shown in the pain score at 24 h between liposomal bupivacaine 133 mg, liposomal bupivacaine 266 mg and bupivacaine 50 mg. They did note, however, a significant difference between 24 and 96 h in favour of liposomal bupivacaine 133 mg, when compared to bupivacaine, and at 96 h, when compared with liposomal bupivacaine 266 mg.^16^

### Surgical techniques

Fourteen studies investigated different surgical techniques.^7–11,19–26,28^ Four RCTs^7,8,19,20^ and three meta-analyses^22,26,28^ specifically explored the analgesic benefits of a minimal invasive approach or percutaneous technique vs. open osteotomy.^7,8,19,20,22,26,28^ Among these three meta-analyses, one of seven articles (371 patients; 4 articles included for our current recommendations; 3 excluded as either no pain scores were reported (*n* = 2) or patient were not randomised (*n* = 1))^22^ and one of nine articles (666 patients: 2 articles included for our current recommendations; 7 excluded for no pain scores reported (*n* = 2), retrospective design (*n* = 4) or patients were not randomised (*n* = 1))^28^ found no analgesic benefit with the minimal invasive approach or percutaneous technique when they were compared with an open technique. However, the third meta-analysis of 22 articles (1415 patients: 4 articles included for our current recommendations; 18 excluded for no pain scores reported (*n* = 4), a retrospective design (*n* = 12) or patients were not randomised (*n* = 2))^26^ found a decrease in the pain score at 2 weeks with the use of minimally invasive surgery compared with an open technique. Interestingly, three more recent RCTs comparing an open technique with a minimally invasive technique found a decrease in the pain score by 1.1 (*n* = 71)^20^ and 1.4 (*n* = 58)^19^ at 24 h and by 2 at hospital discharge (*n* = 50) with the latter technique.^7^ In a further trial, that included 39 patients, no difference in the pain scores were shown at 6 weeks, 3 months, 9 months and 5 years between patients who had percutaneous osteotomy and those who had open osteotomy.^8^

In a meta-analysis of 25 trials (1597 patients: 11 articles included for our current recommendations; 14 excluded for no pain scores reported (*n* = 13) or published in 1993 (*n* = 1)), Dias *et al.* explored different interventions for hallux valgus, including non-invasive treatment, different types of surgery and no treatment.^23^ They found no difference in the pain score between simple and complex osteotomies. Patients who had surgery reported reduced pain scores when compared with medical treatment or no treatment at 12 months.^23^ Milczarek *et al.* found no analgesic benefit of adding an Akin proximal phalanx correction on top of a scarf corrective osteotomy in 155 patients.^10^ In a meta-analysis of 10 trials (685 patients: 4 articles included for our current recommendations; 6 excluded for no pain scores reported (*n* = 5) or published in 1993 (*n* = 1)), Fukushi *et al.* investigated the optimal site for osteotomy and showed no difference between midshaft, proximal or distal osteotomies with respect to analgesic outcomes.^24^

The use of biodegradable magnesium and titanium screws did not lead to a difference in the pain score in a meta-analysis of five trials (301 patients: 2 articles included for our current recommendations; 3 excluded for retrospective design).^25^ Pain scores were similar between groups in three RCTs that compared traditional oscillating saw with piezoelectric tool system (*n* = 34),^11^ topical skin adhesive with nylon sutures (*n* = 84)^9^ and traditional method with 3D printed navigation template technology (*n* = 48).^21^

### Other modalities

After including a total of 90 patients and comparing the flat shoe with the reverse camber shoe, Dearden *et al.* did not find any difference between the pain score at rest and on movement at the postoperative 6 week time point.^6^ Based on the results of 70 patients, Plaass *et al.* concluded that a dynamic splint decreased the pain score at rest but not on movement at 3 months. The authors did not specify, however, whether a multimodal analgesic treatment was prescribed.^13^ Finally, a virtual reality mask for the management of preoperative anxiety did not result in a reduction in the pain score and opioid consumption prior to hospital discharge.^15^

## Discussion

After reviewing 17 RCTs and seven systematic reviews/meta-analysis published since 2019 and following the PROSPECT approach, we have updated our recommendations for analgesia after hallux valgus repair surgery. These recommendations are listed in Table 1. Table 2 details the analgesic interventions that are not recommended for pain management. Table 3 presents the evolution of the recommendations between this updated review and the previously published original review.^2^ The strength of our systematic review stems from the PROSPECT methodology which goes beyond making recommendations based on the simple statistical analysis of the available evidence. We have interpreted the included studies based on the use of a baseline analgesic technique in the control group and balancing the benefits and adverse effects of the intervention as well as assimilating this information in a clinical context (i.e., in the setting of the *surgical procedure*). Overall, the PROSPECT recommendations provide clinicians with supporting arguments for and against the use of analgesic interventions for hallux valgus repair.

**Table 1:** Overall recommendations for pain management in patients undergoing hallux valgus repair surgery. In this updated review, there is one major change: the recommendation of the surgical technique (minimal invasive surgery or percutaneous osteotomy)

**Pharmacological treatments** • Paracetamol combined with a nonsteroidal anti-inflammatory drug or cyclooxygenase (COX)-2 selective inhibitor administered preoperatively or intra-operatively and continued postoperatively • Dexamethasone (systemic), intraoperatively • Opioid for rescue postoperatively
**Anaesthetic and analgesic strategies** • Ankle block with single administration of local anaesthetics as first choice • Local anaesthetic wound infiltration as an alternative
**Surgical procedures** • Minimally invasive surgery or percutaneous osteotomy
**Other modalities** • None

**Table 2:** Analgesic interventions that are not recommended for pain management in patients undergoing hallux valgus repair surgery

	Intervention	Reason for not recommending
*Regional anaesthesia*	Popliteal sciatic nerve block with local anaesthetic and magnesium sulphate	Limited procedure-specific evidence
	Popliteal sciatic nerve block with liposomal bupivacaine	Limited procedure-specific evidence
	Mode of infusion for continuous popliteal sciatic nerve block	Lack of procedure-specific evidence
	Plantar compartment nerve block and fibular nerve block with local anaesthetic	Lack of procedure-specific evidence
	Biodegradable magnesium screws or titanium screws	Lack of procedure-specific evidence
*Surgical techniques*	Ludloff osteotomy guided by preoperative plan with 3D printed navigation	Lack of procedure-specific evidence
	Piezoelectric tool system with microvibrations for distal linear osteotomy	Lack of procedure-specific evidence
	Topical skin adhesive or nylon sutures for surgical incision closure	Lack of procedure-specific evidence
*Other modalities*	Dynamic splint (stretch traction) for hallux valgus correction	Lack of procedure-specific evidence
	Virtual reality mask hypnosis prior to surgery	Lack of procedure-specific evidence
	Rigid sole flat shoe (Darco MedSurg) vs. reverse camber shoe (Darco OrthoWedge) following surgery	Lack of procedure-specific evidence

**Table 3:** Comparison of the recommended interventions for pain management in patients undergoing hallux valgus repair surgery with the previous PROSPECT recommendations

	2020	2025
*Pharmacological treatments*		
Paracetamol	X	X
NSAID/COX-2 specific inhibitor	X	X
Dexamethasone (systemic)	X	X
Opioids	X	X
*Anaesthetic and analgesic strategies*		
Ankle block (first choice choice)	X	X
Wound infiltration (alternative)	X	X
*Surgical procedures*		
Minimal invasive surgery or percutaneous osteotomy		X
*Other modalities*		
None		

In this updated review, the one major change is the recommendation of the surgical technique (minimal invasive surgery or percutaneous osteotomy).

Korwin-Kochanowska *et al.*^2^

The analgesic regimen for hallux valgus repair should include, in the absence of contraindication, paracetamol and a nonsteroidal anti-inflammatory drug or cyclooxygenase-2 selective inhibitor administered preoperatively or intra-operatively and continued postoperatively, along with systemic dexamethasone, and postoperative opioids for rescue analgesia. In this updated review, the one major change is the recommendation on the surgical technique. Previously, we stated that this should be left to the discretion of the surgeon, based on their experience and expertise,^2^ particularly as the data published at that time on a percutaneous technique came from only a single trial.^29^ With a meta-analysis^26^ and three further positive trials,^7,19,20^ however, we now have enough evidence to definitively recommend a minimally invasive approach or percutaneous osteotomy over an open osteotomy.

The contemporary literature confirms the analgesic effect of an ankle block as a first-choice modality, with wound infiltration as an alternative. While a popliteal sciatic nerve block provides similar analgesia to an ankle block,^27,30^ this regional anaesthetic technique is not recommended as it prevents the patient from walking without crutches, impairing functional outcomes. As the ankle block requires several painful injections, we recommend performing the procedure under sedation, as previously described.^30^ That said, two trials performed a popliteal sciatic nerve block when investigating an intervention such as perineural magnesium sulfate^14^ or liposomal bupivacaine.^16^ This is of potential interest. Magnesium sulphate, as a perineural adjunct to local anaesthetics, should probably not be administered as its safety profile has still not been fully and properly addressed.^31,32^ Further, recent publications have now underlined the analgesic value of concomitant dexamethasone with regional anaesthetic techniques. An intraoperative i.v. dose of 0.1 to 0.2 mg kg^−1^ is suggested since it prolongs the analgesia in the postoperative period by a mean duration of 8 h in the presence of a regional anaesthetic technique which has used long-acting local anesthetic^31,32^

While liposomal bupivacaine has been found to decrease the pain score for up to 96 h,^16^ we question the biological plausibility of such analgesia provision.^16^ When compared with 50 mg of bupivacaine, there was no analgesic difference with 266 mg of liposomal bupivacaine at 96 h (*P* = 0.15), whereas a significant analgesic difference was reported with a dose of 133 mg at the same postoperative time (*P* = 0.0012). When compared with 50 mg of bupivacaine, analgesia differences with 133 mg of liposomal bupivacaine were reported at 48 h (*P* < 0.001) and 72 h (*P* < 0.001), but not at 24 h (*P* = 0.68), but there was no report of pain difference at 24, 48 or 72 h with a dose of 266 mg liposomal bupivacaine. In the absence of intraneural injection or a perineural adjunct, it is unexpected that 50 mg of bupivacaine would have an analgesic efficacy that lasted 24 h. A recent meta-analysis which included 27 trials and 2122 patients concluded that liposomal bupivacaine when compared with long-acting local anaesthetics (bupivacaine or ropivacaine) reduced the pain score at rest by 0.9 out of 10 at 24 h and by 0.7 up to 72 h following surgery.^33^ The mean difference of less than 1 out of 10 is less than the minimal clinically important difference and challenges the clinical efficacy of liposomal bupivacaine.^4^

That said, according to the PROSPECT methodology, insufficient evidence was shown for the administration of perineural magnesium sulphate or the use of liposomal bupivacaine, as their analgesic benefits were only found by a single trial, and we therefore do not recommend these. We would also not advocate for more trials in these two fields of analgesic intervention for the previously mentioned reasons.

### Future directions

Future adequately powered trials should assess the effects of analgesic interventions not only on pain, opioid consumption, opioid-related adverse events and complications associated with the intervention, but also on functional outcomes. Moreover, the influence of analgesic interventions on chronic pain and chronic opioid therapy need to be considered. Similarly, the effects of analgesic interventions in high-risk patients (high pain-responders) need to be studied.

### Limitations

The limitations in this review are related to those of the studies included. Firstly, there was considerable heterogeneity between studies such as differing control groups, dosing regimens, methods of administration, and variable time points of pain assessments. Secondly, the small size of most studies has the potential for estimation effect. In addition, the sample sizes of these studies were not sufficient to draw valid conclusions with regard to the safety profile of the analgesic interventions. Thirdly, the analgesic interventions were not always evaluated against a control group that included an optimised multimodal analgesic regimen.

## Conclusion

In conclusion, this review has updated the previous guidelines written by the PROSPECT group. The one important change lies in the recommendation that minimally invasive surgery or percutaneous osteotomy should be used instead of open osteotomy. Contemporary publications confirm the analgesic effect of ankle block as a first-choice modality with wound infiltration as an alternative. The analgesic regimen for hallux valgus repair should include, in the absence of contraindication, paracetamol and a nonsteroidal anti-inflammatory drug or cyclooxygenase-2 selective inhibitor administered preoperatively or intra-operatively and continued postoperatively, along with systemic dexamethasone, and postoperative opioids for rescue analgesia.

## Supplementary Material

Supplemental Digital Content
