# Supplementary material for: Pain management after hallux valgus repair surgery: an updated systematic review and procedure-specific postoperative pain management (PROSPECT) recommendations
Source: Eur J Anaesthesiol. 2025 Oct 21;43(1):11–8. doi: 10.1097/EJA.0000000000002302 (PMC12700695; doi:10.1097/EJA.0000000000002302)
Supplement: Supplemental Digital Content [file ejanet-43-11-s001.docx]

**Search strategies**

**Medline Ovid SP**

(exp Bunion/su OR Metatarsophalangeal Joint/su OR exp Hallux Valgus/su OR ((Bunion* OR post-bunion* OR Hallux OR "Metatarsophalangeal joint*") AND (repair* OR surger* OR operation* OR resection* OR surgical* OR osteotomy OR bunionectomy OR Post-operativ* OR postoperativ* OR post-surgery OR post-surgical* OR postsurgical* OR post-bunion*)).ab,ti,kf.) AND (exp Pain/ OR exp Pain Management/ OR exp Pain Measurement/ OR exp Anesthesia and Analgesia/ OR exp Analgesics/ OR exp Anesthetics/ OR exp Clonidine/ OR exp Pregabalin/ OR exp Ketamine/ OR exp Acetaminophen/ OR exp Gabapentin/ OR exp Cyclooxygenase 2/ OR exp dexamethasone/ OR exp bupivacaine/ OR (pain* OR Analgesi* OR anaesthe* OR anesthe* OR vas OR (visual ADJ1 analog*) OR vrs OR nrs OR (("verbal rating" OR "numeric* rating" OR "analog* pain") ADJ1 scale*) OR ((formalin OR nocicepti* OR tourniquet) ADJ3 test*) OR mcgill OR epidural* OR neuraxial OR intrathecal OR paravertebral OR spinal OR caudal OR intercostal OR interpleural OR infiltration OR ((nerve OR neural OR paravertebral OR field OR peripheral OR ankle) ADJ3 (nerve* OR block*)) OR COX-2 OR ((Cyclo-Oxygenase OR cyclooxygenase) ADJ1 (II OR 2)) OR NSAID* OR ((nonsteroidal OR non-steroidal) ADJ3 anti-inflammator*) OR Paracetamol OR acetaminophen OR clonidine OR opioid* OR ketamine OR corticosteroid* OR gabapentin* OR pregabalin OR dexamethasone* OR betamethasone OR bupivacaine OR levobupivacaine OR "systemic steroid*").ab,ti,kf.) AND ((randomized controlled trial.pt. OR controlled clinical trial.pt. OR randomized.ab. OR placebo.ab. OR drug therapy.fs. OR randomly.ab. OR trial.ab. OR groups.ab.) NOT (exp animals/ not humans.sh.))

**Embase.com**

('hallux valgus'/exp/dm_su OR 'bunionectomy'/exp OR 'bunionette'/exp/dm_su OR 'metatarsal osteotomy'/exp OR 'hallux valgus surgery'/exp OR ((Bunion* OR post-bunion* OR Hallux OR "Metatarsophalangeal joint*") AND (repair* OR surger* OR operation* OR resection* OR surgical* OR osteotomy OR bunionectomy OR Post-operativ* OR postoperative* OR post-surgery OR post-surgical* OR postsurgical* OR post-bunion*)):ab,ti,kw) AND ('pain'/exp OR 'pain measurement'/exp OR 'pain assessment'/exp OR 'analgesia'/exp OR 'anesthesiological procedure'/exp OR 'analgesic agent'/exp OR 'clonidine'/exp OR 'anesthetic agent'/exp OR 'corticosteroid'/exp OR 'cyclooxygenase 2'/exp OR 'bupivacaine'/exp OR (pain* OR Analgesi* OR anaesthe* OR anesthe* OR vas OR (visual NEXT/1 analog*) OR vrs OR nrs OR (("verbal rating" OR "numeric* rating" OR "analog* pain") NEXT/1 scale*) OR ((formalin OR nocicepti* OR tourniquet) NEAR/3 test*) OR mcgill OR epidural* OR neuraxial OR intrathecal OR paravertebral OR spinal OR caudal OR intercostal OR interpleural OR infiltration OR ((nerve OR neural OR paravertebral OR field OR peripheral OR ankle) NEXT/3 (nerve* OR block*)) OR COX-2 OR ((Cyclo-Oxygenase OR cyclooxygenase) NEXT/1 (II OR 2)) OR NSAID* OR ((nonsteroidal OR non-steroidal) NEXT/3 anti-inflammator*) OR Paracetamol OR acetaminophen OR clonidine* OR opioid* OR ketamine OR corticosteroid* OR gabapentin* OR pregabalin OR dexamethasone* OR bupivacaine OR levobupivacaine OR "systemic steroid*" OR betamethasone):ab,ti,kw) AND (((random* OR factorial* OR crossover* OR cross NEXT/1 over* OR placebo* OR doubl* NEXT/1 blind* OR singl* NEXT/1 blind* OR assign* OR allocat* OR volunteer*):de,ab,ti OR 'crossover procedure'/exp OR 'double blind procedure'/exp OR 'randomized controlled trial'/exp OR 'single blind procedure'/exp) NOT ([animals]/lim NOT [humans]/lim))

**CINAHL EBSCO**

(MH "Metatarsophalangeal Joint/SU" OR MH "Hallux Valgus/SU" OR MH "Bunionectomy" OR TI(((Bunion* OR Hallux OR "Metatarsophalangeal joint*") AND (repair* OR surger* OR operation* OR resection* OR surgical*)) OR "chevron osteotomy") OR AB(((Bunion* OR Hallux OR "Metatarsophalangeal joint*") AND (repair* OR surger* OR operation* OR resection* OR surgical*)) OR "chevron osteotomy")) AND (MH "Pain Measurement" OR MH "Pain Management" OR MH "Pain+" OR MH "Analgesics+" OR MH "Narcotics+" OR MH "Anesthetics+" OR MH "Gabapentin" OR MH "Clonidine" OR MH "Dexamethasone" OR MH "Betamethasone" OR TI(Analgesi* OR anaesthe* OR anesthe* OR vas OR (visual NEXT/1 analog*) OR vrs OR nrs OR (("verbal rating" OR "numeric* rating" OR "analog* pain") NEXT/1 scale*) OR ((formalin OR nocicepti* OR tourniquet) NEAR/3 test*) OR mcgill OR epidural* OR neuraxial OR intrathecal OR paravertebral OR spinal OR caudal OR intercostal OR interpleural OR infiltration OR ((nerve OR neural OR paravertebral OR field OR peripheral OR ankle) NEXT/3 (nerve* OR block*)) OR COX-2 OR ((Cyclo-Oxygenase OR cyclooxygenase) NEXT/1 (II OR 2)) OR NSAID* OR ((nonsteroidal OR non-steroidal) NEXT/3 anti-inflammator*) OR Paracetamol OR acetaminophen OR clonidine* OR opioid* OR ketamine OR corticosteroid* OR gabapentin* OR pregabalin OR dexamethasone* OR bupivacaine OR levobupivacaine OR "systemic steroid*" OR betamethasone) OR AB(Analgesi* OR anaesthe* OR anesthe* OR vas OR (visual NEXT/1 analog*) OR vrs OR nrs OR (("verbal rating" OR "numeric* rating" OR "analog* pain") NEXT/1 scale*) OR ((formalin OR nocicepti* OR tourniquet) NEAR/3 test*) OR mcgill OR epidural* OR neuraxial OR intrathecal OR paravertebral OR spinal OR caudal OR intercostal OR interpleural OR infiltration OR ((nerve OR neural OR paravertebral OR field OR peripheral OR ankle) NEXT/3 (nerve* OR block*)) OR COX-2 OR ((Cyclo-Oxygenase OR cyclooxygenase) NEXT/1 (II OR 2)) OR NSAID* OR ((nonsteroidal OR non-steroidal) NEXT/3 anti-inflammator*) OR Paracetamol OR acetaminophen OR clonidine* OR opioid* OR ketamine OR corticosteroid* OR gabapentin* OR pregabalin OR dexamethasone* OR bupivacaine OR levobupivacaine OR "systemic steroid*" OR betamethasone)) AND (MH (randomized controlled trials OR double‐blind studies OR single‐blind studies OR random assignment OR pretest‐posttest design OR cluster sample ) OR TI (randomised OR randomized) OR AB random* OR TI trial OR ( (MH (sample size) AND AB (assigned OR allocated OR control))) OR MH (placebos OR crossover design OR comparative studies) OR AB ((control W5 group) OR (cluster W3 RCT) OR PT (randomized controlled trial))) NOT ((MH animals+ OR MH (animal studies) OR TI (animal model*)) NOT MH (human))

**Cochrane Central Register of Controlled Trials Wiley**

(((Bunion* OR post-bunion* OR Hallux OR (Metatarsophalangeal NEXT joint*)) AND (repair* OR surger* OR operation* OR resection* OR surgical* OR osteotomy OR bunionectomy OR (Post NEXT operativ*) OR postoperative* OR post-surgery OR (post NEXT surgical*) OR postsurgical* OR (post NEXT bunion*))):ab,ti,kw) AND ((pain* OR Analgesi* OR anaesthe* OR anesthe* OR (visual NEXT/1 analog*) OR vrs OR nrs OR (("verbal rating" OR (numeric*NEXT rating*) OR (analog* NEXT pain)) NEXT/1 scale*) OR ((formalin OR nocicepti* OR tourniquet) NEAR/3 test*) OR mcgill OR epidural* OR neuraxial OR intrathecal OR paravertebral OR spinal OR caudal OR intercostal OR interpleural OR infiltration OR ((nerve OR neural OR paravertebral OR field OR peripheral OR ankle) NEXT/3 (nerve* OR block*)) OR COX-2 OR ((Cyclo-Oxygenase OR cyclooxygenase) NEXT/1 (II OR 2)) OR NSAID* OR ((nonsteroidal OR non-steroidal) NEXT/3 (anti NEXT inflammator*)) OR Paracetamol OR acetaminophen OR clonidine* OR opioid* OR ketamine OR corticosteroid* OR gabapentin* OR pregabalin OR dexamethasone* OR bupivacaine OR levobupivacaine OR (systemic NEXT steroid*) OR betamethasone):ab,ti,kw)

**Web of Science – Core Collection**

TS=((((Bunion* OR post-bunion* OR Hallux OR (Metatarsophalangeal NEAR/1 joint*)) NEAR (repair* OR surger* OR operation* OR resection* OR surgical* OR osteotomy OR bunionectomy OR (Post NEAR/1 operativ*) OR postoperative* OR post-surgery OR (post NEAR/1 surgical*) OR postsurgical* OR (post NEAR/1 bunion*)))) AND ((pain* OR Analgesi* OR anaesthe* OR anesthe* OR vas OR (visual NEAR/1 analog*) OR vrs OR nrs OR (("verbal rating" OR (numeric* NEAR/1 rating*) OR (analog* NEAR/1 pain)) NEAR/1 scale*) OR ((formalin OR nocicepti* OR tourniquet) NEAR/2 test*) OR mcgill OR epidural* OR neuraxial OR intrathecal OR paravertebral OR spinal OR caudal OR intercostal OR interpleural OR infiltration OR ((nerve OR neural OR paravertebral OR field OR peripheral OR ankle) NEAR/2 (nerve* OR block*)) OR COX-2 OR ((Cyclo-Oxygenase OR cyclooxygenase) NEAR/1 (II OR 2)) OR NSAID* OR ((nonsteroidal OR non-steroidal) NEAR/2 (anti NEAR/1 inflammator*)) OR Paracetamol OR acetaminophen OR clonidine* OR opioid* OR ketamine OR corticosteroid* OR gabapentin* OR pregabalin OR dexamethasone* OR bupivacaine OR levobupivacaine OR (systemic NEAR/1 steroid*) OR betamethasone))) AND TS=(randomised OR randomized OR randomisation OR randomization OR placebo* OR (random* AND (allocat* OR assign*)) OR (blind* AND (single OR double OR treble OR triple)))

**Table S1.** Summary of key results from studies evaluating systemic analgesics, analgesics adjuncts, regional anaesthesia, and surgical procedures used to support the recommended interventions in patients after hallux valgus repair surgery.

| **Study** | **Study design** | **Pain scores** | **Cumulative opioid consumption** | **Basic analgesics Administered** |
| --- | --- | --- | --- | --- |
| **REGIONAL ANAESTHESIA** | | | | |
| ***Ankle nerve block*** | | | | |
| Ozhan et al. 2020^12^ | All under general anaesthesia  No ankle block (n = 55)  vs.  Ankle block (n = 55) | Decreased at 12 h in ankle block group (2.4 vs. 5.3; *P* = 0.001) | Reduced in ankle block group (*P* = 0.001)  Longer time to first rescue analgesic in ankle block group (*P* = 0.001) | Paracetamol, diclofenac, PCA with tramadol, pethidine |
| ***Ankle block, peri-incisional infiltration*** | | | | |
| Su et al. 2019^18^ | All under general anaesthesia  No block (n = 26)  vs.  Ankle block (n = 25)  vs.  Peri-incisional infiltration  (n = 24) | Decreased in recovery room (*P* < 0.01) and at 6 h (3.2 vs. 0.4 vs. 1.3; *P* = 0.001) in ankle block group  No difference at 12 h  (*P* = 0.09), 24 h (*P* = 0.44), and 36 h (*P* = 0.87) | Reduced at 6 h (*P* < 0.001) and 12 h (*P* = 0.008) in ankle block group | Paracetamol, diclofenac, PCA with fentanyl, |
| ***Ankle block, peri-incisional block, popliteal sciatic nerve block*** | | | | |
| Ravanbod et al. 2022^27^ | Ankle block  vs.  Peri-incisional infiltration  vs.  Popliteal sciatic nerve block  Systematic review and meta-analysis (n = 5 articles; 459 patients) | Decreased at 24 h in ankle block group and peri-incisional infiltration group when compared to no regional anaesthesia (1.3 vs. 3.0; *P* < 0.001)  Decreased at 6 h in ankle block group when compared with peri-incisional infiltration or no regional anaesthesia (*P* < 0.001)  No difference between ankle block and popliteal sciatic nerve block groups  (*P* = 0.12) | Reduced opioid consumption in ankle block group (*P* < 0.001) | Not applicable |
| **SURGICAL TECHNIQUE** | | | | |
| ***Minimal invasive or percutaneous technique*** | | | | |
| Kaufmann et al. 2020^8^ | Percutaneous chevron osteotomy (n = 19)  vs.  Open chevron osteotomy (n = 20) | NS (pain scores measured at 6 weeks, 9 months and 5 years) | Not specified | Not specified |
| Torrent et al. 2021^19^ | Minimally invasive scarf osteotomy (n = 30)  vs.  Open scarf osteotomy (n = 28) | Decreased at 24 h in minimally invasive surgery group (2.3 vs. 3.7; *P* = 0.03) | NS | Paracetamol, dexketoprofen,  tramadol |
| Dragosloveanuet al. 2022^7^ | Percutaneous chevron osteotomy (n = 24)  vs.  Open chevron osteotomy (n = 26) | Decrease at hospital discharge (2.5 vs. 4.5; *P* < 0.001), 3 weeks (1.4 vs. 2.8; *P* < 0.001), 6 weeks (0.4 vs. 2.0; *P* < 0.001), and 6 months (0.2 vs. 0.8; *P* = 0.004) in percutaneous osteotomy group | Not specified | Not specified |
| Yoon et al. 2024^20^ | Percutaneous distal chevron osteotomy (n = 36)  vs.  Open distal chevron osteotomy (n = 35) | Decreased at POD1 in percutaneous osteotomy group (4.2 vs. 5.3 *P* = 0.019) | Not specified | Not specified |
| Ji et al. 2022^26^ | Minimally invasive surgery  vs.  Open surgery  Systematic review and meta-analysis (n = 22; 1415 patients) | Decreased at 2 weeks in minimally invasive surgery group (*P* < 0.001)  No difference at 6 months (*P* = 0.11) | Not specified | Not applicable |
| Alimy et al. 2022^22^ | Minimal invasive surgery  vs.  Open surgery  Systematic review and meta-analysis (n = 7; 371 patients) | NS (pain scores measured at last follow-up available) | Not specified | Not applicable |
| Singh et al. 2020^28^ | Minimally invasive surgery  vs.  Open surgery  Systematic review and meta-analysis (n = 9; 666 patients) | NS (pain scores measured at last follow-up available) | Not specified | Not applicable |

MA, meta-analysis; NS, no significant difference between groups; POD, postoperative day; PCA, patient-controlled analgesia; RA, regional anaesthesia; SR, systematic review.

**Table S2.** Summary of key results from studies evaluating systemic analgesics, analgesics adjuncts, regional anaesthesia and surgical procedures used to support the interventions that are not recommended for analgesic benefit in patients having hallux valgus repair surgery.

| **Study** | **Study design** | **Pain scores** | **Cumulative opioid consumption** | | **Basic analgesics Administered** |  |
| --- | --- | --- | --- | --- | --- | --- |
| **REGIONAL ANAESTHESIA** | | | | | | |
| ***Popliteal sciatic block, compartment block, fibular block*** | | | | | | |
| Swisser et al. 2024^17^ | Popliteal sciatic nerve block (n = 29)  vs.  Popliteal sciatic nerve block + plantar compartment nerve block + fibular nerve blocks (n = 30) | NS (pain scores measured from recovery room to POD3) | NS | | Paracetamol, ketoprofen |  |
| Pulitano et al. 2024^14^ | Popliteal sciatic nerve block (n = 25)  vs.  Popliteal sciatic nerve block + magnesium sulphate (n = 25) | Decreased from 12 to 24 h in magnesium sulphate group (2 vs. 6; *P* < 0.001) | Reduced in magnesium sulphate group (*P* < 0.001) | | Paracetamol, morphine |  |
| Breebaart et al. 2021^5^ | Continuous infusion 5 ml h^-1^ + patient-controlled boluses 6 ml 30 min^-1^ (n = 19)  vs.  Automated intermittent boluses of 0.1 ml h^-1^ and 9.8 ml boluses every 2 h + patient-controlled boluses 6ml 30 min^-1^ (n = 23) | NS (pain scores measured at 48 h) | NS | | Paracetamol, ketorolac, tramadol |  |
| Schwartz et al. 2024^16^ | *Part A*  Liposomal bupivacaine 266 mg (n = 22)  or  Liposomal bupivacaine 133 mg (n = 22)  vs.  Bupivacaine 50 mg (n = 66)  *Part B*  Liposomal bupivacaine 133 mg (n = 81)  vs.  Bupivacaine 50 mg  (n = 82) | *Part A*  Decreased at 48 h (*P* < 0.001), 72 h (*P* < 0.001) and 96 h (*P* = 0.0012) in liposomal bupivacaine 133 mg group, but not at 24 h (*P* = 0.68)  No difference between liposomal bupivacaine 266 mg and bupivacaine group (*P* = 0.20)  *Part B*  No difference at 24 h (*P* = 0.68)  Reduced from 24 to 96 h with liposomal bupivacaine 133 mg (*P* < 0.00001) | Reduced at 96 h in liposomal bupivacaine 133 mg group (*P* < 0.00001) | | Paracetamol, celecoxib, morphine or hydromorphone |  |
| **SURGICAL TECHNIQUE** | | | | | | |
| ***Open surgery, minimally invasive*** | | | | | | |
| Milczarek et al. 2021^10^ | Scarf corrective osteotomy (n = 87)  vs.  Scarf corrective osteotomy + Akin proximal phalanx correction (n = 58) | NS (pain scores measured at 2 years) | Not specified | Paracetamol, tramadol | |  |
| Fukushi et al. 2022^24^ | Midshaft  vs.  Proximal  vs.  Distal  Systematic review and meta-analysis (n = 10; 685 patients) | NS (time measurement not specified) | Not specified | Not applicable | |  |
| Dias et al. 2024^23^ | Surgical interventions  vs.  Other treatment  Systematic review and meta-analysis (n = 25; 1597 patients) | NS (pain scores measured at 12 months) | Not specified | Not applicable | |  |
| Fu et al. 2023^25^ | Biodegradable magnesium screws  vs.  Titanium screws  Systematic review and meta-analysis (n = 5; 266 patients) | NS (pain scores measured at last follow-up available) | Not specified | Not applicable | |  |
| Lewis et al. 2021^9^ | Topical skin adhesive (n = 42)  vs.  Nylon sutures (n = 42) | NS (pain scores measured at 2 and 6 weeks) | Not specified | Not specified | |  |
| Mosca et al. 2021^11^ | Traditional oscillating saw (n = 17)  vs.  Piezoelectric tool system (n = 17) | NS (pain scores measured at 1 year) | Not specified | Not specified | |  |
| Zhao et al. 2023^21^ | Traditional method (n = 24)  vs.  3D printed navigation template technology (n = 24) | NS after surgery and at 1month | Not specified | Not specified | |  |
| ***Other*** | | | | | |  |
| Dearden et al. 2019^6^ | Flat shoe (Darco MedSurg) (n = 43)  vs.  Reverse camber shoe (Darco OrthoWedge) (n = 47) | NS (pain scores measured at 6 weeks) | Not specified | Paracetamol, codeine, tramadol | |  |
| Plaass et al. 2019^13^ | No splint (n = 34)  vs.  Dynamic splint (n = 36) | Decreased at rest in dynamic splint group (2.4 vs. 3.9; *P* = 0.014) at 3 months  No difference on walking (*P* = 0.099) | Not specified | Not specified | |  |
| Rougereau et al. 2023^15^ | No virtual reality mask (n = 30)  vs.  Virtual reality mask (n = 30) | NS (pain scores measured prior to hospital discharge) | NS | Paracetamol, nefopam, tramadol, morphine, ketamine | |  |

MA, meta-analysis; NS, no significant difference between groups; POD, postoperative day; PCA, patient-controlled analgesia; RA, regional anaesthesia; SR, systematic review.

**Table S3.** List of articles excluded and reasons for exclusion.

| **Study** | **Comparators** | **Reason for exclusion** |
| --- | --- | --- |
| Armstrong et al. 2022 | Abobotulinumtoxin A 300U or 500U *vs.* placebo | Not commercialised  Phase 2 trial |
| Ammar et al. 2021 | Preclinical discovery and development of oliceridine for the treatment of post-operative pain | No randomised controlled trial, systematic review or meta-analysis |
| Bahar et al. 2021 | Patients who were shown preoperative foot photos after hallux valgus surgery *vs.* patients who were not shown the photos | No randomised controlled trial, systematic review or meta-analysis |
| Baker et al. 2022 | Plantar plate repair through either dorsal *vs.* plantar approach | Mixed surgeries |
| Baravarian et al. 2023 | Posthoc analysis of abobotulinumtoxin A *vs.* placebo | Not commercialized |
| Baumann et al. 2023 | Metatarsophalangeal arthrodesis *vs.* other surgeries | No pain score between 0-10 |
| Beard et al. 2021 | Oliceridine *vs.* morphine | No pain score between 0-10 |
| Bello et al. 2023 | Reduce sesamoid group *vs.* non reduce sesamoid group after distal chevron osteotomy | No randomised controlled trial, systematic review or meta-analysis |
| Biz et al. 2021 | Sciatic-femoral blocks *vs.* ankle block | No randomised controlled trial, systematic review or meta-analysis |
| Bourn et al. 2022 | Bupivacaine/meloxicam extended release *vs.* bupivacaine HCL or bupivacaine extended release or meloxicam extended release or placebo | Not commercialized |
| Carter et al. 2020 | To compare intravenous non-opioid analgesics | Trial on basic analgesia |
| Chang et al. 2022 | Plantar plate repair using standard operative instruments | No randomised controlled trial, systematic review or meta-analysis |
| Choi et al. 2024 | Proximal *vs.* distal chevron osteotomy | No pain score between 0-10 |
| Cohena et al. 2021 | Chevron osteotomy for the correction of hallux valgus | No randomised controlled trial, systematic review or meta-analysis |
| Coster et al. 2021 | Osteotomy *vs.* cheilectomy | No randomised controlled trial, systematic review or meta-analysis |
| Daniels et al. 2019 | Ibuprofen and paracetamol *vs.* ibuprofen or paracetamol or placebo | Trial on basic analgesia |
| Desai et al 2024 | Intravenous dexamethasone *vs.* perineural dexamethasone | Mixed surgeries |
| Di caprio et al. 2023 | Interposition arthroplasties *vs.* other alternatives | No pain score between 0-10 |
| Drobnic et al. 2021 | Surgical treatment of hallux rigidus using a novel implant | No randomised controlled trial, systematic review or meta-analysis |
| Ebina et al. 2020 | Modified osteotomy with medial capsule interposition *vs.* no medial capsule interposition | No randomised controlled trial, systematic review or meta-analysis |
| Ferguson et al. 2021 | Intravenous single ibuprofen dose *vs.* placebo or active control | Trial on basic analgesia |
| Filippi et al. 2020 | Complications after metatarsal osteotomies | No randomised controlled trial, systematic review or meta-analysis |
| Finney et al. 2019 | Persistent opioid use after surgery *vs.* not persistent opioid use | No randomised controlled trial, systematic review or meta-analysis |
| Fleischer et al. 2020 | Isolated second Weil metatarsal osteotomy *vs.* Weil metatarsal osteotomy and plantar plate repair | No randomised controlled trial, systematic review or meta-analysis |
| Franco de la torre et al. 2023 | Intrarectal tapentadol *vs.* placebo | Trial on basic analgesia |
| Fung et al. 2020 | Orthosis *vs.* non orthosis | No randomised controlled trial, systematic review or meta-analysis |
| Galli et al. 2021 | Cheilectomy alone *vs.* cheilectomy with umbilical cord allograft | No trial registry |
| Ghioldi et al. 2022 | Learning curve of a surgeon with the 30 first surgeries *vs.* learning curve with the 30 next surgeries | No randomised controlled trial, systematic review or meta-analysis |
| Glazebrook et al. 2019 | 5-year prospective follow-up of patients who received a polyvinyl alcohol synthetic cartilage implant hemiarthroplasty *vs.* first metatarsophalangeal joint arthrodesis | Mixed surgeries |
| Hanslik-schnabel et al. 2022 | First metatarsophalangeal joint arthrodesis using human allogenic cortical bone screw *vs.* standard arthrodesis technique | No randomised controlled trial, systematic review or meta-analysis |
| Harraser et al. 2023 | Minimal invasive osteotomy with one screw *vs.* minimal invasive with two screws | No randomised controlled trial, systematic review or meta-analysis |
| Hartenbach et al. 2022 | Interdigital approach *vs.* medial transarticular approach for lateral release combined with scarf osteotomy | No pain score between 0-10 |
| Herteleer et al. 2024 | Popliteal sciatic block *vs.* combined plantar compartment and peroneal blocks | No randomised controlled trial, systematic review or meta-analysis |
| He et al. 2021 | First metatarsophalangeal joint arthrodesis *vs.* arthroplasty for rheumatoid forefoot deformity | Mixed surgeries |
| Hernandez-Castillejo et al. 2021 | Open *vs.* percutaneous surgery | No pain score between 0-10 |
| Horita et al. 2022 | Resection arthroplasty *vs.* shortening oblique osteotomy of lesser metatarsal combined with arthrodesis of the first metatarsophalangeal joint | No randomised controlled trial, systematic review or meta-analysis |
| Hutchins et al. 2023 | Liposomal bupivacaine 266mg *vs.* liposomal bupivacaine and Bupivacaine HCL vs. bupivacaine HCL | No full text article |
| Ilfeld et al. 2021 | Nerve electrical stimulation *vs.* placebo | Mixed surgeries |
| Jackson et al. 2024 | Opioid consumption in different surgeries | No randomised controlled trial, systematic review or meta-analysis |
| Joo et al. 2021 | Synthetic cartilage implant hemiarthroplasty *vs.* arthrodesis | No randomised controlled trial, systematic review or meta-analysis |
| Korean registry et al. 2020 | Percutaneous distal metatarsal osteotomy *vs.* open distal chevron osteotomy | No full text article |
| Koh et al. 2023 | Cheilectomy and subchondroplasty *vs.* cheilectomy | No randomised controlled trial, systematic review or meta-analysis |
| Kuik et al. 2021 | Safety and efficacy of medial plate in the first metatarsophalangeal joint arthrodesis | No randomised controlled trial, systematic review or meta-analysis |
| Kush et al. 2024 | Degenerative changes at the tibial sesamoid-first metatarsal joint *vs.* not degenerative changes | No randomised controlled trial, systematic review or meta-analysis |
| Langford et al. 2024 | Co-cristal tramadol-celecoxib *vs.* tramadol or celecoxib | No randomised controlled trial, systematic review or meta-analysis |
| Lewis et al. 2022 | Function and quality life outcomes in female with hallux valgus | No randomised controlled trial, systematic review or meta-analysis |
| Lisacek-Kiosoglous et al. 2022 | Minimally invasive *vs.* open surgery | No full text article |
| Li et al. 2022 | Intravenous parecoxib *vs.* placebo or active control | Trial on basic analgesia |
| Ling et al. 2020 | Weight-bearing regime at 2 weeks *vs.* 6 weeks postoperatively | No pain score between 0-10 |
| Lucar-Lopez et al. 2024 | Distal rotation metatarsal osteotomy *vs.* regular approach | No pain score between 0-10 |
| Luo et al. 2020 | Subparaneural injection *vs.* extraparaneural injection for popliteal sciatic nerve block | Mixed surgeries |
| Mehraban et al. 2021 | Standard 2-step skin antiseptic preparation of alcohol followed by chlorhexidine/alcohol *vs.* povidone-iodine soak and scrub followed by that same standard 2-step skin preparation | Mixed surgeries |
| Menz et al. 2022 | Multifaceted, non-surgical intervention *vs.* advice and self-management alone | No pain score between 0-10 |
| Miranda et al. 2021 | Complications on percutaneous hallux valgus surgery *vs.* other techniques | No pain score between 0-10 |
| Munteanu et al. 2024 | Non-surgical treatment *vs.* placebo, no treatment or other treatment | Mixed surgeries |
| Niu et al. 2023 | Oliceridine *vs.* opioids | Mixed surgeries |
| Oosterbos et al. 2022 | Conservative *vs.* surgical treatment of foot drop in peroneal nerve entrapment | Mixed surgeries |
| Pappas et al. 2024 | Midterm outcomes open *vs.* minimal invasive procedures | No randomised controlled trial, systematic review or meta-analysis |
| Paterson et al. 2019 | Different treatment of metatarsophalangeal joint osteoarthritis | No full text article |
| Phimister et al. 2023 | Targeting a peripheral sodium channel to treat pain | No randomised controlled trial, systematic review or meta-analysis |
| Radovic et al. 2020 | Botulinum injection in addition to electrical stimulation *vs.* original method | No randomised controlled trial, systematic review or meta-analysis |
| Rajan et al. 2022 | Isolated hallux valgus correction *vs.* concomitant hammertoe correction | No randomised controlled trial, systematic review or meta-analysis |
| Rivero et al. 2019 | Weil osteotomy *vs.* distal metatarsal mini-invasive osteotomy | No pain score between 0-10 |
| Rooney et al. 2023 | Opioid free *vs.* opioid containing postoperative protocol | Mixed surgeries |
| Rogero et al. 2019 | Postoperative opioid consumption across different type of hallux valgus surgeries | No randomised controlled trial, systematic review or meta-analysis |
| Scheidt et al. 2022 | Lessert toe deformity correction with proximal joint arthrodesis *vs.* joint resection arthroplasty | Mixed surgeries |
| Sever et al. 2019 | Correction with longitudinal capsulorrhaphy *vs.* inverted L type capsulorrhaphy | No randomised controlled trial, systematic review or meta-analysis |
| Shafer et al. 2024 | Vocacapsaicin 0.30, 0.15, or 0.05 mg.ml^-1^ *vs.* placebo | Not commercialized |
| Shadid et al. 2019 | Chlorhexidine, alcohol 0.5%/70% *vs.* iodine, alcohol 1%/70% | No pain score between 0-10 |
| Sieloff et al. 2023 | Scarf osteotomy *vs.* scarf osteotomy with additional procedure | No pain score between 0-10 |
| Singla et al. 2020 | Tramadol 50mg or 25mg *vs.* placebo | Trial on basic analgesia |
| Sommer et al. 2024 | Pain research in 2023: towards understanding chronic pain | No randomised controlled trial, systematic review or meta-analysis |
| Spindler et al. 2024 | Compare surgical procedure *vs.* surgical procedure or procedure for different stages of hallux valgus | No pain score between 0-10 |
| Stamenkovic et al. 2021 | Updates on wound infiltration use for pain management | No randomised controlled trial, systematic review or meta-analysis |
| Stibolt et al. 2019 | Partial *vs.* total joint replacement | Mixed surgeries |
| Tsai et al. 2021 | Degenerative changes at the metatarsal head sesamoid articulation during hallux valgus correction | No randomised controlled trial, systematic review or meta-analysis |
| Veracruz-Galvez et al. 2022 | Influence of sesamoid position after scarf osteotomy for hallux valgus on patient-reported outcome | No randomised controlled trial, systematic review or meta-analysis |
| Viscusi et al. 2019 | Tapentadol immediate release *vs.* opioids or placebo | Mixed surgeries |
| Viscusi et al. 2019 | bupivacaine and meloxicam in biochronomerpolymer technology *vs.* bupivacaine HCl or placebo | Not commercialized |
| Viscusi et al. 2024 | Co-crystal of celecoxib and tramadol *vs.* celecoxib or tramadol or placebo | Trial on basic analgesia |
| Viscusi et al. 2019 | Oliceridine *vs.* placebo or morphine | Not commercialized |
| Wang et al. 2019 | Revolving scarf osteotomy *vs.* double metatarsal osteotomy | No randomised controlled trial, systematic review or meta-analysis |
| Wong et al. 2023 | Plantar load before *vs.* after hallux valgus surgery | No pain score between 0-10 |
| Xu et al. 2024 | Ropivacaine extended-release *vs.* ropivacaine HCl or placebo | Not commercialized |
| Ying et al. 2021 | Comparative of different conservative treatment | No pain score between 0-10 |
| Yip et al. 2022 | Extended-release bupivacaine and meloxicam *vs.* bupivacaine HCL or placebo | No randomised controlled trial, systematic review or meta-analysis |
| Kong et al. 2021 | Combined ropivacaine and betamethasone *vs.* intravenous analgesia pump | Foreign language |
